# Influence of subject discontinuation on long-term nonvertebral fracture rate in the denosumab FREEDOM Extension study

**DOI:** 10.1186/s12891-017-1520-6

**Published:** 2017-04-27

**Authors:** Jonathan D. Adachi, Henry G. Bone, Nadia S. Daizadeh, Paula Dakin, Socrates Papapoulos, Peyman Hadji, Chris Recknor, Michael A. Bolognese, Andrea Wang, Celia J. F. Lin, Rachel B. Wagman, Serge Ferrari

**Affiliations:** 1 McMaster University, 501-25 Charlton Ave E., Hamilton, ON L8N 1Y2 Canada; 2 Michigan Bone and Mineral Clinic, 22201 Moross Rd, Detroit, MI 48236 USA; 3 Amgen Inc., One Amgen Ctr Dr., Thousand Oaks, CA 91320 USA; 4 Leiden University Medical Center, Albinusdreef 2, 2333 ZA Leiden, Netherlands; 5 Krankenhaus Nordwest, Steinbacher Hohl 2-26, 60488 Frankfurt am Main, Germany; 6 United Osteoporosis Centers, 2350 Limestone Pkwy, Gainesville, GA 30501 USA; 7 Bethesda Health Research Center, 10215 Fernwood Rd Ste 40, Bethesda, MD 20817 USA; 8 Geneva University Hospital, Rue Gabrielle-Perret-Gentil 4, 1205 Genève, Switzerland

**Keywords:** Denosumab, Osteoporosis, Selection bias, Extension study, FREEDOM

## Abstract

**Background:**

Denosumab treatment for up to 8 years in the FREEDOM study and Extension was associated with low fracture incidence. It was not clear whether subjects who discontinued during the study conduct had a higher risk of fracture than those who remained enrolled, thereby underestimating the true fracture risk for the entire trial cohort. Thus, we explored the influence of early withdrawals on nonvertebral fracture incidence during the Extension study.

**Methods:**

To understand the potential effect of depletion of susceptible subjects on fracture incidence, we first evaluated subject characteristics in patients who were enrolled in the Extension vs those who were not. We subsequently employed a Kaplan-Meier multiple imputation (KMMI) approach to consider subjects who discontinued as if they remained enrolled with a 0%, 20%, 50%, and 100% increase in fracture risk compared with participants remaining on study.

**Results:**

Extension enrollees were generally similar to nonparticipants in median age (71.9 and 73.1 years, respectively), mean total hip bone mineral density T-score (–1.9 and –2.0, respectively), and probability of fracture risk by Fracture Risk Assessment Tool (FRAX^®^) at FREEDOM baseline (16.9% and 17.7% for major osteoporotic fracture and 6.7% and 7.4% for hip fracture, respectively). When we assumed a doubled fracture risk (100% increase) after discontinuation in KMMI analyses, nonvertebral fracture rate estimates were only marginally higher than the observed rates for both the crossover group (10.32% vs 9.16%, respectively) and the long-term group (7.63% vs 6.63%, respectively).

**Conclusion:**

The observation of continued denosumab efficacy over 8 years of treatment was robust and does not seem to be explained by depletion of susceptible subjects.

**Trial registration:**

ClincalTrials.gov registration number NCT00523341; registered August 30, 2007

**Electronic supplementary material:**

The online version of this article (doi:10.1186/s12891-017-1520-6) contains supplementary material, which is available to authorized users.

## Background

Osteoporosis is a chronic disease requiring continuing treatment, and information on the long-term safety and efficacy of treatments is therefore essential. In the 3-year FREEDOM study, treatment with the receptor activator of nuclear factor kappa-B ligand inhibitor denosumab significantly reduced the incidence of vertebral, nonvertebral, and hip fractures compared with placebo [1]. To assess the safety and efficacy of denosumab for up to 10 years, the FREEDOM study was extended. FREEDOM subjects who were eligible and desired to participate in the Extension were transitioned to denosumab if they had formerly received placebo (crossover group) or continued denosumab treatment (long-term group). Denosumab treatment for up to 8 years was associated with low fracture incidence, continued bone mineral density (BMD) increases, and an adverse event profile similar to what was previously reported [2–6].

The interpretation of the continued low incidence of fractures in the Extension study is limited by the lack of a placebo control group as a reference for the incidence of fracture in untreated subjects. Therefore, it is conceivable that changes in study population characteristics might affect interpretation of efficacy and safety outcomes. Subjects may discontinue participation in an osteoporosis clinical trial for a variety of reasons. The nature of long-term follow-up with prescribed visits may prove to be a hindrance in an aging population. In addition, the occurrence of fracture or adverse events may discourage participation. As in all long-term studies, subject discontinuation has the potential to bias results through the depletion of available susceptible subjects.

The primary aim of the present analyses [7, 8] was to evaluate the possible influence of early withdrawals on nonvertebral fracture incidence during the Extension study. For this evaluation, we used a Kaplan-Meier multiple imputation (KMMI) approach [9]. We created four scenarios, where we assumed different increases in fracture risk for the subjects who discontinued the Extension study early compared to the participants who remained on study. We then compared results from the KMMI scenarios to those obtained from the Extension primary analyses.

## Methods

### Study design

As described previously, FREEDOM was a phase 3, multicenter, randomized, double-blind, placebo-controlled study with a duration of 3 years [1]. Subjects were randomized to receive either denosumab 60 mg or placebo by subcutaneous (SC) injection every 6 months. In the Extension, all subjects were to receive denosumab 60 mg SC every 6 months for a total of 7 years (Additional file 1). Description of the Extension study has been published elsewhere [2, 3] and is summarized briefly. This manuscript includes data from the first 5 years of the Extension, representing up to 8 years of denosumab treatment for the long-term group and up to 5 years of denosumab treatment for the crossover group.

Key eligibility criteria to participate in the Extension required that the patients:

- did not discontinue investigational medicine and completed the 36-month visit in the FREEDOM study,
- did not miss more than one dose of investigational medicine during the FREEDOM study, and
- provided consent to participate in the Extension.

A full list of criteria is shown in Additional file 2. We note that T-score thresholds, part of the eligibility criteria in the FREEDOM study to identify postmenopausal women with osteoporosis, were not required for subjects to be eligible for enrollment in the Extension.

### Statistical analysis

Subject characteristics were assessed descriptively over time in subjects who enrolled in the FREEDOM study, those who enrolled in the Extension, and those who remained in the Extension study at the end of year 8. Characteristics of subjects at the FREEDOM study baseline were also assessed descriptively in subjects who enrolled in the Extension and in subjects who did not participate in the Extension. In addition, the most frequent reasons for study discontinuation were compared descriptively between the FREEDOM study and its Extension.

The effect of denosumab on long-term nonvertebral fracture risk was assessed by Kaplan-Meier estimate of time to first nonvertebral fracture over the first 5 years of the Extension (up to 8 years of denosumab treatment). Cumulative estimates and 95% confidence intervals are reported at the yearly time points. Vertebral fractures are not included in these analyses, as they were ascertained by radiography at specified intervals and were not amenable to time-to-event analysis.

Sensitivity analyses were undertaken to assess the robustness of the observed Kaplan-Meier estimates of nonvertebral fracture incidence over time. The goal of these sensitivity analyses was to evaluate the possible impact of unknown fracture status (outcome) for subjects who withdrew from the study before experiencing an incident nonvertebral fracture. These analyses employed a KMMI approach for sensitivity analyses of time-to-event data with possibly informative censoring [9]. Informative censoring occurs when subjects who discontinued are either more or less likely to experience the specific event than the remaining individuals in the future. In the current analysis, the specific event of interest is nonvertebral fracture. The KMMI approach imputes fracture status for the discontinued subjects during their unobserved remaining time as if they continued to be followed until the end of the study. A unique feature of this KMMI approach is allowance for the incorporation of various assumed increases in nonvertebral fracture risk for discontinued subjects after study discontinuation relative to the fracture risk of subjects remaining on study when imputing fracture status. Multiple imputation data sets were analyzed individually with the conventional Kaplan-Meier method, and the results from these separate analyses were then combined using the method of Rubin [10].

We chose four different scenarios of fracture risk—increases of 0%, 20%, 50%, and 100% in fracture risk after discontinuation for the subjects who withdrew before experiencing a nonvertebral fracture compared with the subjects who remained in the trial, with the highest assumption (ie, 100%) representing a doubled fracture risk after withdrawal. As judged by baseline characteristics and Fracture Risk Assessment Tool (FRAX^®^), a 100% increase in fracture risk (doubling of the risk) was determined to be a reasonable “worst-case” scenario for these participants, because subjects who enrolled in the FREEDOM trial were not a particularly high-risk population [1, 11], and the subjects had few comorbid conditions. For each scenario, 50 imputations were performed.

## Results

### Subject disposition through extension year 5

**Fig. 1:**
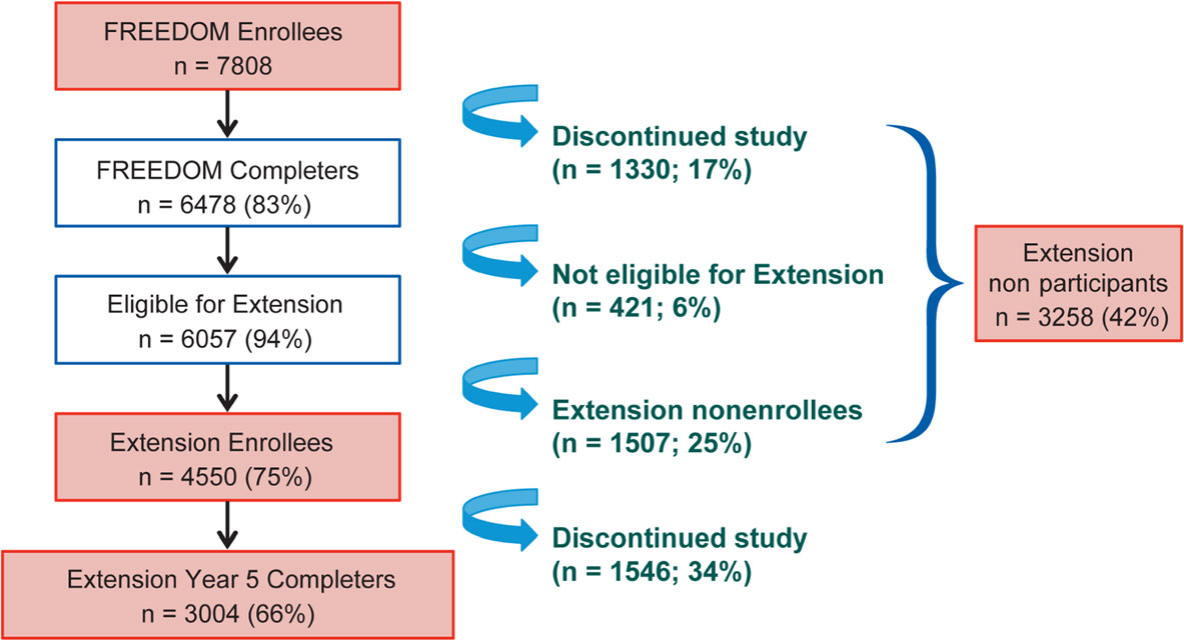
Evolution of subject sample size over time

The numbers of subjects participating in the FREEDOM study and its Extension are shown in Fig. 1. Eighty-three percent of subjects completed the FREEDOM study (6478/7808), of whom 8% (550) were ineligible to participate in the Extension; the most common reason was missing more than one dose of investigational medicine during the FREEDOM study, *n* = 421. Of those who were eligible (*n* = 5928), 1378 (23%) chose not to participate in the Extension. Thus, 77% of eligible subjects (*n* = 4550) enrolled in the Extension study. Hence, of the original 7808 subjects who participated in FREEDOM, 3258 (42%) subjects did not participate in the Extension (Extension nonparticipants). At the end of Extension year 5, 3004 subjects (66% of the Extension baseline population or 38% of the original FREEDOM population) remained enrolled in the study.

### Extension enrollees vs nonparticipants

FREEDOM baseline characteristics for Extension enrollees and nonparticipants are shown in Table 1. The mean age was slightly higher for Extension nonparticipants (73.1 years) compared with Extension enrollees (71.9 years). The proportion of subjects ≥ 70 years of age was slightly higher in Extension nonparticipants (77%) compared with Extension enrollees (71%).

**Table 1:** Characteristics of Extension enrollees and nonparticipants at FREEDOM baseline

	Placebo (*N* = 3906)		Denosumab (*N* = 3902)		Combined (*N* = 7808)	
	Extension nonparticipants (*N* = 1699)	Extension enrollees (*N* = 2207)	Extension nonparticipants (*N* = 1559)	Extension enrollees (*N* = 2343)	Extension nonparticipants (*N* = 3258)	Extension enrollees (*N* = 4550)
Age, years						
Mean (SD)	73.1 (5.3)	71.8 (5.1)^a^	73.0 (5.4)	71.9 (5.0)^a,b^	73.1 (5.4)	71.8 (5.0)
Median (range)	73 (60–91)	72 (60–90)	73 (60–89)	72 (60–90)	73 (60–91)	72 (60–90)
Age group, *n* (%)						
≥ 70 years	1307 (77)	1571 (71)	1200 (77)	1672 (71)	2507 (77)	3243 (71)
≥ 75 years	612 (36)	624 (28)^a^	573 (37)	662 (28)^a,b^	1185 (36)	1286 (28)
≥ 80 years	203 (12)	143 (6)	178 (11)	144 (6)	381 (12)	287 (6)
Prior fracture, *n* (%)						
Prior vertebral	430 (25)	485 (23)^a^	370 (24)	559 (24)^a,b^	800 (25)	1044 (23)
Prior nonvertebral^c^	526 (31)	651 (29)	461 (30)	702 (30)^b^	987 (30)	1353 (30)
BMD T-score, mean (SD)						
Lumbar spine	–2.8 (0.7)	–2.8 (0.7)^a^	–2.8 (0.7)	–2.8 (0.7)^a,b^	–2.8 (0.7)	–2.8 (0.7)
Total hip	–2.0 (0.8)	–1.9 (0.8)^a^	–1.9 (0.8)	–1.9 (0.8)^a,b^	–2.0 (0.8)	–1.9 (0.8)
FRAX^®^ probability,^d^ %, mean (SD)						
Hip	7.4 (7.7)	6.6 (7.3)	7.5 (8.0)	6.8 (7.2)	7.4 (7.8)	6.7 (7.3)
Major osteoporotic	17.8 (9.8)	16.8 (9.7)	17.6 (9.9)	17.0 (9.6)	17.7 (9.8)	16.9 (9.6)

*BMD* bone mineral density, *FRAX* ^*®*^ Fracture Risk Assessment Tool, *SD* standard deviation

^a^Published in Papapoulos et al, 2012 [2]

^b^Published in Ferrari et al, 2015 [5]

^c^At age ≥ 55 years

^d^10-year probability of fracture calculated with femoral neck BMD

**Fig. 2:**
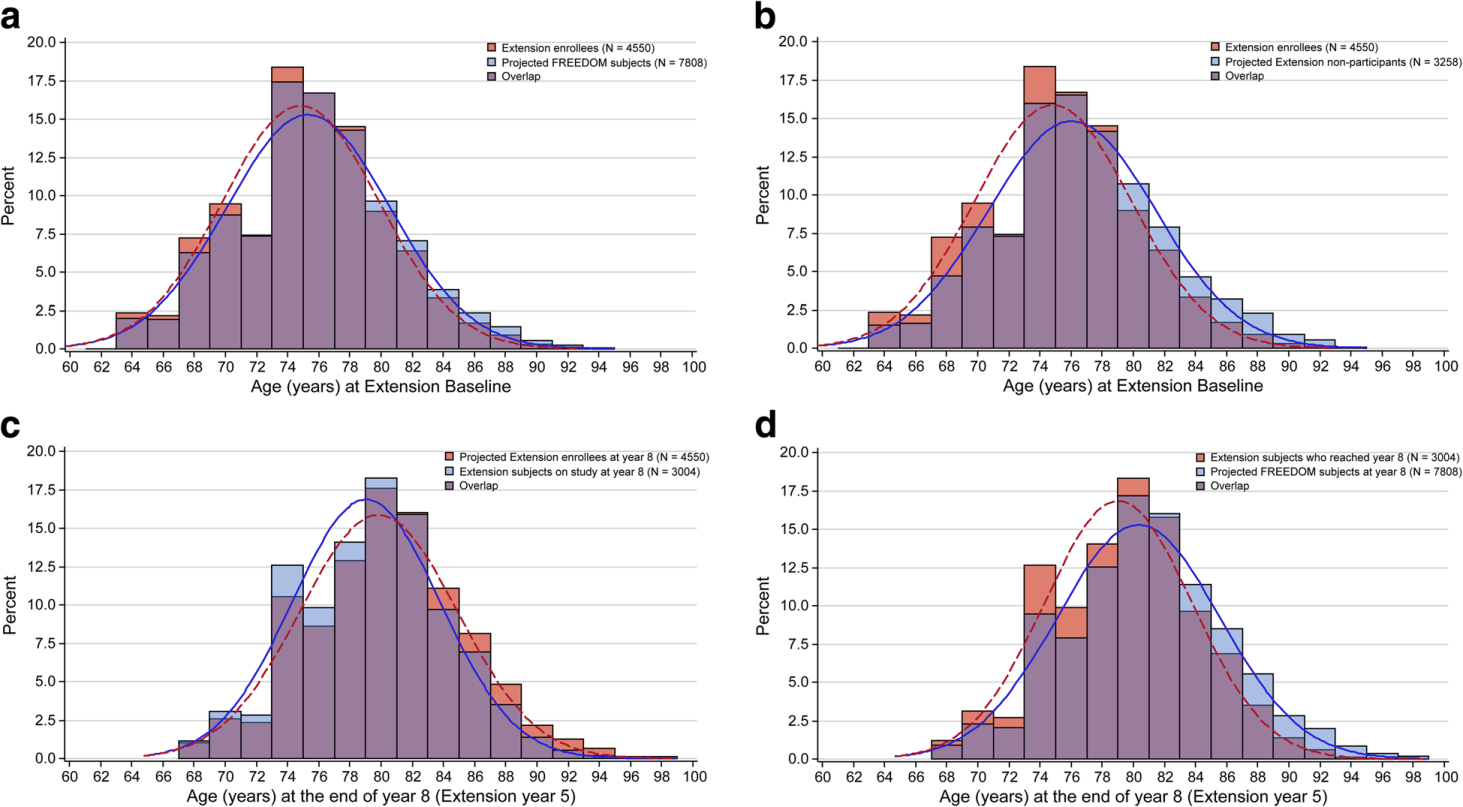
Age distribution. **a** Observed age of Extension enrollees vs projected age of FREEDOM population at Extension baseline and overlap between the groups. *Lines* represent smoothed age distribution. **b** Observed age of Extension enrollees vs projected age of Extension nonparticipants at Extension baseline and overlap between the groups. *Lines* represent smoothed age distribution. **c** Projected age of Extension enrollees at year 8 vs observed age of subjects who reached year 8 and overlap between the two groups. *Lines* represent smoothed age distribution. **d** Observed age of subjects who reached the end of year 8 vs projected age of FREEDOM population at the end of year 8; overlap between the groups. *Lines* represent smoothed age distribution

Figure 2a compares the observed age distribution of the Extension enrollees at Extension baseline with the projected age distribution of the entire FREEDOM population if all subjects enrolled into the Extension, and shows a considerable overlap between the age distributions of these two populations. The observed age distribution for Extension enrollees and the projected age distribution for Extension nonparticipants at Extension baseline also overlapped, although the Extension nonparticipant population included older subjects (Fig. 2b).

No substantial differences were observed for other key risk factors for fracture between the enrollees and nonparticipants. Prior nonvertebral fracture incidence was the same (30%) between the Extension enrollees and nonparticipants, while BMD at the hip was similar, albeit slightly higher, in Extension enrollees compared with nonparticipants (Table 1). The age of subjects increased in a manner generally consistent with the study length, whereby at the start of the FREEDOM study, the median age was 72.0 years; at the end of 3 years, median age was 75.0 years; and after 8 years, median age was 79.0 years (Table 2). Figure 2c compares the projected age of all Extension enrollees at the end of year 8 to the observed age of the subset of subjects who reached the end of year 8. The difference between the two age distributions reflects a small percentage loss of some of the oldest subjects from the Extension study population. Figure 2d compares the age of the population remaining at the end of year 8 with the projected age of the FREEDOM population at the end of year 8, showing that the proportion of older subjects who were lost from the study over 8 years remained relatively small. Among the 3004 subjects who remained in the study at the end of year 8, 47% were ≥ 80 years old, compared with a projected 53% of Extension enrollees or 56% of the FREEDOM subjects. When we examined the mean 10-year FRAX^®^ probability of fracture at FREEDOM baseline, the fracture probability was similar for Extension enrollees compared with Extension nonparticipants (6.7% vs 7.4% for a hip fracture and 16.9% vs 17.7% for a major osteoporotic fracture, with femoral neck BMD included in the calculations). Similarly, the baseline FRAX^®^ scores for subjects who completed year 8 of the Extension study were somewhat lower than those who discontinued during the FREEDOM or Extension studies. The subjects who discontinued had a relative increase in fracture risk of 7% to 26% (absolute increase between 1.1% and 1.8%; Additional file 3).

**Table 2:** Comparison of subject characteristics over time

	FREEDOM baseline		Extension baseline		End of year 8	
	Placebo (*N* = 3906)	Denosumab (*N* = 3902)	Crossover denosumab (*N* = 2207)	Long-term denosumab (*N* = 2343)	Crossover denosumab (*N* = 1462)	Long-term denosumab (*N* = 1542)
Age, years						
Mean (SD)	72.3 (5.2)^a^	72.3 (5.2)^a^	74.8 (5.1)^b^	74.9 (5.0)^b^	79.0 (4.8)	79.1 (4.7)
Median (range)	72 (60–91)	72 (60–90)	75 (63–93)	75 (63–93)	79 (68–98)	79 (68–94)
Age group, *n* (%)						
≥ 70 years	2878 (74)	2872 (74)	1823 (83)	1974 (84)	1420 (97)	1505 (98)
≥ 75 years	1236 (32)^a^	1235 (32)^a^	1151 (52)^b^	1258 (54)^b^	1160 (79)	1255 (81)
≥ 80 years	346 (9)	322 (8)	379 (17)	407 (17)	676 (46)	737 (48)
Prior fracture, *n* (%)						
Prior vertebral	915 (23)^a^	929 (24)^a^	551 (25)^b,c^	573 (24)^b,c^	NA	NA
Prior nonvertebral^d^	1177 (30)	1163 (30)	754 (34)^c^	780 (33)^c^	NA	NA
BMD T-score, mean (SD)						
Lumbar spine	–2.8 (0.7)^a^	–2.8 (0.7)^a^	–2.8 (0.8)^b^	–2.1 (0.8)^b^	–1.9 (0.9)	–1.6 (0.9)
Total hip	–1.9 (0.8)^a^	–1.9 (0.8)^a^	–1.9 (0.8)^b^	–1.5 (0.8)^b^	–1.5 (0.8)	–1.3 (0.8)

*BMD* bone mineral density, *NA* not applicable (because the prior fractures cannot be defined at the end of the treatment), *SD* standard deviation

^a^Published in Cummings et al, 2009 [1]

^b^Published in Papapoulos et al, 2012 [2]

^c^Includes incident fractures during FREEDOM

^d^At age ≥ 55 years

### Reasons for early withdrawal

The most frequent reasons for study discontinuation were similar between the FREEDOM study and the Extension (Table 3). Less than 15% patients had an adverse event listed as the reason for discontinuation, and no more than 1% in either group had any particular adverse event listed as the reason for discontinuation. Subjects who experienced a fracture during the study were more likely to remain on study compared with those who did not: during the FREEDOM study, discontinuation rates in the placebo group were 12% in patients with an incident fracture and 19% in those without an incident fracture and 17% vs 16%, respectively, in the denosumab group. During the Extension, 28% of patients with an incident fracture withdrew compared with 35% of those without an incident fracture.

**Table 3:** Top reasons for discontinuation during FREEDOM and during the Extension

	FREEDOM (Years 1–3)		Extension year 5 (Years 1–5)	
	Placebo (*N* = 3906)	Denosumab (*N* = 3902)	Crossover denosumab (*N* = 2207)^a^	Long-term denosumab (*N* = 2343)^a^
Reasons for discontinuation, *n* (%)	700 (18)	630 (16)	745 (34)	801 (34)
Consent withdrawn	403 (10)	344 (9)	325 (15)	337 (14)
Other	20 (1)	32 (1)	170 (8)^b^	184 (8)^b^
Adverse event	81 (2)	93 (2)	93 (4)	115 (5)
Death	78 (2)	62 (2)	64 (3)	80 (3)
Lost to follow-up	57 (1)	57 (1)	60 (3)	46 (2)

^a^Published in Papapoulos et al 2012 [2]

^b^Includes discontinuation after a protocol amendment to extend the Extension for an additional 5 years, which was associated with an increased discontinuation rate at the end of Extension year 2

### Kaplan-Meier estimates of nonvertebral fractures

**Fig. 3:**
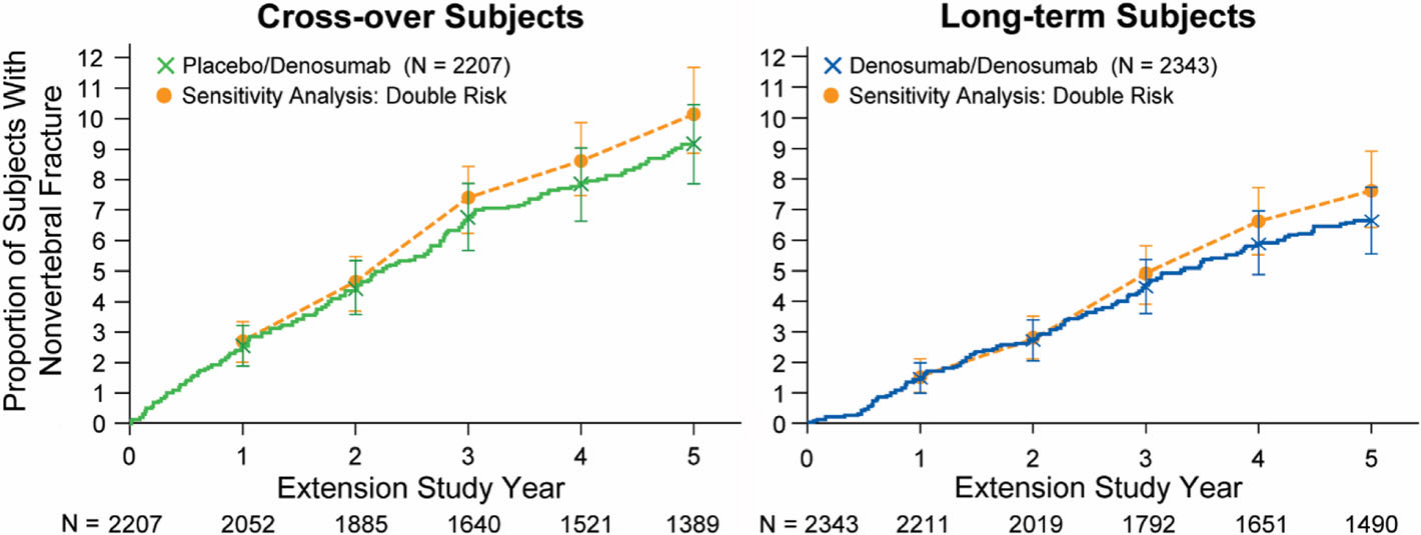
Observed fracture incidence vs Kaplan-Meier estimated fracture incidence in sensitivity analyses. We compared the observed cumulative fracture incidence in crossover and long-term denosumab-treated subjects up to Extension year 5 (year 8 overall) vs the Kaplan-Meier estimated fracture incidence using a multiple imputation approach with the assumption that subjects who withdrew had double the fracture risk after study discontinuation compared with those who remained on the study. *N* is the number of subjects who remained at risk at the beginning of each year

The cumulative Kaplan-Meier estimates of incidence of nonvertebral fractures during the Extension were 2.55%, 4.45%, 6.77%, 7.84%, and 9.16% for the crossover group and 1.49%, 2.72%, 4.48%, 5.92%, and 6.63% for the long-term group at Extension years 1, 2, 3, 4, and 5, respectively (Fig. 3 and Table 4). The results based on the KMMI approach assuming a range of increases in fracture risk after study withdrawal (0%, 20%, 50%, and 100%) for subjects who withdrew prematurely relative to the subjects with continued follow-up are presented in Table 4. When the assumed increase in fracture risk was 0%, subjects who prematurely withdrew and those who continued follow-up had the same tendency to experience a nonvertebral fracture in the future (corresponding to the observed original data results). Furthermore, the results obtained using this KMMI approach for the 20%, 50%, and 100% (or double) increased risk of fracture remained similar to the observed data for all scenarios assessed (Table 4).

**Table 4:**
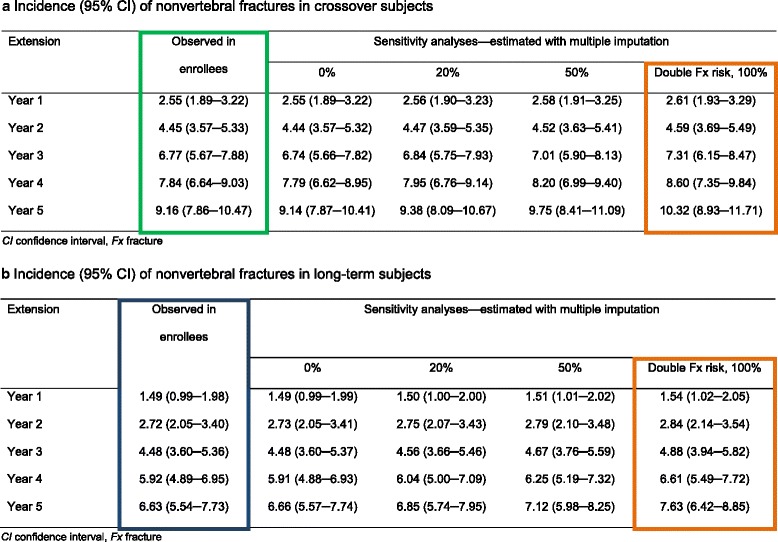
Sensitivity analyses for incidence of nonvertebral fractures in crossover **(a)** and long-term **(b)** subjects

A multiple imputation approach was used with varying assumptions (0, 20, 50, and 100% [ie, double]) of increased fracture risk after study discontinuation for subjects who withdrew early from the Extension relative to subjects who remained in the study. Colored boxes correspond to data plotted in Fig. 3

## Discussion

It is of concern that study subjects who choose to withdraw from participation in an osteoporosis clinical trial may have been more likely to experience an incident fracture on study, a situation often described as depletion of susceptible subjects. Preferential drop-out of susceptible study subjects, potentially resulting in retention of a cohort of “healthier” participants (a so-called “healthy cohort effect”), could influence fracture rates observed in any long-term osteoporosis study, including the Extension study. Results of the first 5 years of the Extension (up to 8 years of denosumab for the long-term group and up to 5 years for the crossover group) have shown that longer treatment with denosumab provides continued BMD gains without therapeutic plateau and sustained low fracture incidence [2, 3, 6]. The present analysis attempted to evaluate whether these results reflected continued benefit from denosumab treatment or were the result of a loss of subjects susceptible to fracture over time within the Extension study.

To further understand the implication of early withdrawal of subjects on fracture outcomes during the Extension, we employed a KMMI method for time-to-event data. This analysis was designed to assess the robustness of the nonvertebral fracture results obtained if the subjects who withdrew early before experiencing an incident nonvertebral fracture had remained on study. The KMMI approach used here is an alternative to the traditional clinical trial approach, wherein the status observed upon premature study withdrawal is analyzed (or carried forward) as the final outcome of the study.

The cumulative KMMI estimates of nonvertebral fracture incidence obtained over time remained low and similar to the observed data, even when we assumed that the subjects who withdrew early from the Extension study without an incident fracture had double the fracture risk after study withdrawal compared with the subjects who stayed on study. The baseline mean FRAX^®^ probability for subjects who withdrew early was somewhat higher than the baseline mean FRAX^®^ probability of subjects who continued through year 8, corresponding to a “worst-case” relative increase in fracture risk of 26%. Consequently, a 100% increase, or doubling, in fracture risk is a reasonable “worst-case” scenario for the subjects enrolled in the FREEDOM trial. Therefore, the observation of continued denosumab efficacy throughout the Extension study cannot be explained by early withdrawal of subjects with increased fracture risk.

The sensitivity analysis presented here is one potential method for estimating the impact of early withdrawals on a study outcome measure. A virtual twin analysis [12], conducted after subjects competed 2 years of the Extension study [2], employed a completely different analytical method but led to similar conclusions. This provides further confidence that the observed low fracture incidence over time is the result of denosumab treatment.

We found that Extension nonparticipants had some very slight differences from Extension enrollees for some baseline characteristics. As anticipated, there was a loss of some of the oldest subjects (age ≥ 80 years) during the Extension because of comorbidities and mortality associated with older age. Nevertheless, nearly half of the participants at the end of year 8 were ≥ 80 years old, similar to the proportion projected if the entire FREEDOM population had remained on study through year 8 without adjustment for expected mortality. Further, we demonstrated that preferential loss of subjects who experienced an incident fracture did not occur as we had hypothesized.

## Conclusions

These results show that the observed fracture incidence in the Extension study cannot be explained by depletion of susceptible subjects or, put another way, retention of a healthy cohort of participants. Rather, the results suggest that the low nonvertebral fracture incidence reflects the effect of long-term denosumab treatment, in the context of an aging population.

## Additional files

Additional file 1: Design of FREEDOM and the Extension study. *Q6M* once every 6 months, *SC* subcutaneous. (DOCX 28 kb)

Additional file 2: Eligibility criteria for the Extension study. (DOC 143 kb)

Additional file 3: FREEDOM baseline FRAX^®^ scores for subjects who continued through year 8 compared with those who did not continue through year 8. The 10-year probability of fracture calculated with femoral neck BMD. *BMD* bone mineral density, *FRAX* ^*®*^ Fracture Risk Assessment Tool, *SD* standard deviation. (DOC 29 kb)

Additional file 4: Institutional review boards and ethics committees for the Extension study. (DOC 120 kb)

## Abbreviations

BMD: Bone mineral density
CI: Confidence interval
FRAX^®^: Fracture Risk Assessment Tool
Fx: Fracture
KMMI: Kaplan-Meier multiple imputation
NA: Not applicable
Q6M: Once every 6 months
SC: Subcutaneous
SD: Standard deviation
