# Supplementary material for: Influence of subject discontinuation on long-term nonvertebral fracture rate in the denosumab FREEDOM Extension study
Source: BMC Musculoskelet Disord. 2017 Apr 27;18:174. doi: 10.1186/s12891-017-1520-6 (PMC5408481; doi:10.1186/s12891-017-1520-6)
Supplement: Supplementary file 1 — Design of FREEDOM and the Extension study. Q6M once every 6 months, SC subcutaneous. (DOCX 28 kb) [file 12891_2017_1520_MOESM1_ESM.docx]

Adachi et al

**Additional File 1:** Design of FREEDOM and the Extension study. *Q6M* once every 6 months, *SC* subcutaneous


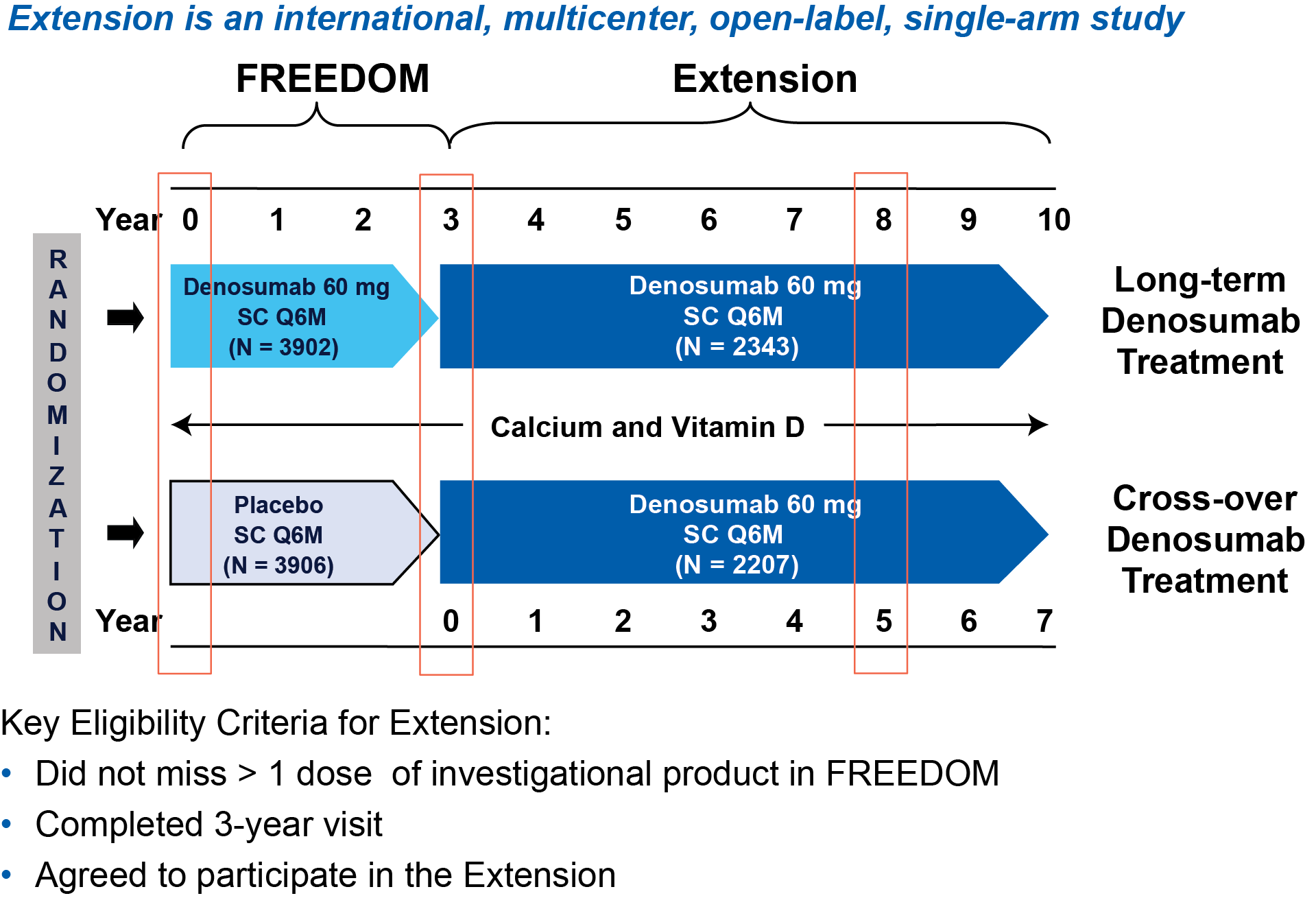


**Reference**

Papapoulos S, Chapurlat R, Libanati C, Brandi ML, Brown JP, Czerwinski E, et al. Five years of denosumab exposure in women with postmenopausal osteoporosis: results from the first two years of the FREEDOM extension. J Bone Miner Res. 2012;27(3):694-701.
