# Supplementary material for: Influence of subject discontinuation on long-term nonvertebral fracture rate in the denosumab FREEDOM Extension study
Source: BMC Musculoskelet Disord. 2017 Apr 27;18:174. doi: 10.1186/s12891-017-1520-6 (PMC5408481; doi:10.1186/s12891-017-1520-6)
Supplement: Supplementary file 2 — Eligibility criteria for the Extension study. (DOC 143 kb) [file 12891_2017_1520_MOESM2_ESM.doc]

**Additional file 2:** Eligibility criteria for the Extension study

| Inclusion criteria | Exclusion criteria |
| --- | --- |
| - Signed consent form and agreed to receive denosumab every 6 months - Did not discontinue investigational product and had the 36-month visit of the FREEDOM study - Gave consent prior to the 24-month visit of the Extension to continue the Extension for 5 more years up to a total of 7 years | - Missed two or more doses of investigational medicine during the FREEDOM study - Permanently nonambulatory (use of an assistive device such as cane or walker was permitted) - Any disorder that, in the opinion of the investigator, may have compromised the ability to give written informed consent and/or comply with study procedures - Developed sensitivity to mammalian cell–derived drug products - Unable to tolerate calcium supplementation - Receiving another investigational medicine - Current use of the following osteoporosis agents: bisphosphonates, calcitonin, fluoride, parathyroid hormone, selective estrogen receptor modulators, systemic oral or transdermal estrogen (except vaginal preparations and estrogen creams), strontium, or tibolone |
