# Supplementary material for: Influence of subject discontinuation on long-term nonvertebral fracture rate in the denosumab FREEDOM Extension study
Source: BMC Musculoskelet Disord. 2017 Apr 27;18:174. doi: 10.1186/s12891-017-1520-6 (PMC5408481; doi:10.1186/s12891-017-1520-6)
Supplement: Supplementary file 3 — FREEDOM baseline FRAX® scores for subjects who continued through year 8 compared with those who did not continue through year 8. The 10-year probability of fracture calculated with femoral neck BMD. BMD bone mineral density, FRAX ® Fracture Risk Assessment Tool, SD standard deviation. (DOC 29 kb) [file 12891_2017_1520_MOESM3_ESM.doc]

**Additional file 3:** FREEDOM baseline FRAX® scores for subjects who completed year 8 of the Extension study compared with those who discontinued during the FREEDOM or Extension studies

| FRAX® probability, %, mean (SD) | Crossover denosumab  (*N* = 2207) | | | Long-term denosumab  (*N* = 2343) | | |
| --- | --- | --- | --- | --- | --- | --- |
|  | Subjects discontinuing the Extension study  (*N* = 745) | Subjects completing year 8 of the Extension study  (*N* = 1462) | Risk increase in subjects who discontinued relative to those who completed (%) | Subjects discontinuing the Extension study (*N* = 801) | Subjects completing year 8 of the Extension study (*N* = 1542) | Risk increase in subjects who discontinued relative to those who completed (%) |
| Hip fracture | 7.7 (8.5) | 6.1 (6.5) | 26% | 7.5 (7.8) | 6.4 (6.9) | 17% |
| Major osteoporotic fracture | 18.0 (10.5) | 16.2 (9.2) | 11% | 17.7 (9.9) | 16.6 (9.4) | 7% |

10-year probability of fracture calculated with femoral neck BMD. *BMD* bone mineral density; *FRAX®* Fracture Risk Assessment Tool, *SD* standard deviation
