# Supplementary material for: Influence of subject discontinuation on long-term nonvertebral fracture rate in the denosumab FREEDOM Extension study
Source: BMC Musculoskelet Disord. 2017 Apr 27;18:174. doi: 10.1186/s12891-017-1520-6 (PMC5408481; doi:10.1186/s12891-017-1520-6)
Supplement: Supplementary file 4 — Institutional review boards and ethics committees for the Extension study. (DOC 120 kb) [file 12891_2017_1520_MOESM4_ESM.doc]

**Additional file 4: Institutional review boards and ethics committees for the Extension study**

| Barwon Health Human Research Ethics Committee |
| --- |
| Bioiatriki |
| Cashmere Radiology |
| CEIC Fundacio de Gestio Sanitaria Hospital de la Santa Creu i Sant Pau |
| CEIC Hospital Universitari i Politecnic La Fe |
| CEIC Hospital Universitario Doctor Peset |
| CEIC Hospital Universitario La Paz |
| CEIC Hospital Universitario Reina Sofia |
| CEIC Hospital Universitario San Cecilio |
| CEIC Hospital Universitartio de La Princesa |
| Center for Clinical and Basic Research Pardubice, as |
| Centralny Szpital Kliniczny Ministerstwa Spraw Wewnetrznych w Warszawie |
| Centre hospitalier deL Universite Laval |
| Centrum Diagnostyki Obrazowej Fantom Spzoo |
| Centrum Medyczne Grupowa Praktyka Lekarska spolka jawna |
| Centrum Medyczne Ogrodowa |
| Clinix Imagerie Medicale et Intervention la Cite |
| Collegium Medicum Uniwersytetu Jagiellonskiego |
| Comitato Etico ASL 3 Genovese di Genova |
| Comitato Etico dell' Azienda Ospedaliera-Universitaria Vittorio Emanuele, Ferrarotto, Santo Bambino |
| Comitato Etico dell Azienda Umberto I di Roma |
| Comitato Etico per la Sperimentazione Clinica dei Medicinali dell A.O. Universita Careggi |
| Comitato Etico per la Sperimentazione Clinica dei Medicinali dell A.O. Universitaria Senese di Siena |
| Comitato Etico per la Sperimentazione Clinica dei Medicinali dell'AOU Integrata di Verona |
| Comitato Etico per la Sperimentazione Dell’ Azienda Ospedaliera di Padova |
| Comite Coordinador de Etica de la Investigacion Biomedica de Andalucia CCEIBA |
| Comite d Ethique Institut Jules Bordet |
| Comité de Docencia e Investigación - Instituto de Investigaciones Metabólicas |
| Comite de Etica de la Facultad de Medicina de la UNAL y Hospital Universitario Dr.Jose Eleuterio Gon |
| Comite de Etica de Revision Institucional para Ensayos en Farmacologia Clinica (CE TIEMPO) |
| Comité de Ética del Centro de Osteopatías Médicas |
| Comite de Etica del Hospital Bernardette |
| Comite de Etica del Instituto Jaliscience de Investigación Clínica |
| Comite de Etica em Pesquisa do Centro Integrado de Atencao a Saude |
| Comitê de Ética em Pesquisa do Hospital Agamenon Magalhaes PE |
| Comite de Etica Em Pesquisa Do Hospital Heliopolis |
| Comite de Etica em Pesquisa do Hospital Pro Cardiaco |
| Comite de Etica em Pesquisa em Seres Humanos da Clinicas da Universidade Federal do Parana |
| Comite de Etica en Investigacion del Centro Medico Dalinde |
| Comite de Investigacion para Estudios en Humanos |
| Comite de Protection des Personnes Sud Est II - Hopital Hotel Dieu |
| Comite d'Ethique Centre Hospitalier Universitaire de Liege |
| Comité Independiente de Ética para Ensayos en Farmacologia Clínica FEFyM Prof Dr Luis Maria Zieher |
| Commissie Medische Ethiek van de Universitaire Ziekenhuizen KU Leuven |
| Commission cantonale d'éthique de la recherche sur l'être humain |
| Commission d Ethique Biomedicale Hospitalo-Facultaire de l UCL |
| Creighton University Institutional Review Board |
| Den Videnskabsetiske Komité for Region Hovedstaden |
| Dorevitch Pathology |
| DXA Mediekos Labor sro Hornicka poliklinika Osteologicke pracoviste Ostrava |
| East Midlands - Derby 1 Research Ethics Committee |
| EC for Clinical Trials on Medical Products |
| Egeszsegugyi Tudomanyos Tanacs, Klinikai Farmakologiai Etikai Bizottsaga |
| Electronic Tomography of Radiology |
| Ethics Committee for Multicenter Trials |
| Ethics Committee of Institute for Treatment and Rehabilitation Niska Banja Nis |
| Ethics Committee of Institute of Rheumatology |
| Ethics Committee of NURCH |
| Ethics Committee Slovenska Medicinska Univerzita |
| Ethikkommission der Stadt Wien |
| Ethik-Kommission Sächsische LÄK |
| Eticka komise Fakultni nemocnice Plzen |
| Eticka komise Krajska Zdravotni as - Nemocnice Chomutov oz |
| Eticka Komise Nemocnice Ceske Budejovice |
| Eticka komise pro multicentricke klinicke hodnoceni, Fakultni nemocnice v Motole |
| Eticka Komise Revmatologickeho Ustavu |
| Eticka Komisia FNsP FD Roosevelta |
| Eticka komisia Nemocnica Kosice-Saca as, I sukromna nemocnica |
| Eticka komisia Univerzitnej nemocnice Bratislava, Nemocnica Ruzinov |
| Eticka komisia Univerzitnej nemocnice Bratislava, Nemocnica sv Cyrila a Metoda |
| Euromedica - Kyanous Stavros Laboratory |
| Exempla IRB |
| Fakultna Nemocnica s poliklinikou FD Roosevelta Banska Bystrica |
| False Creek Surgical Centre |
| Gabinet Diagnostyki i Leczenia Osteoporozy prof dr habn med Wojciech Pluskiewicz |
| General Hospital of Attica Kat Research Laboratory of Muscoskeletal Diseases |
| Health Ethics Committee |
| Helsingin Yliopistollinen Eettinen Toimikunta |
| HUS Ethics Committee (HUS Tutkimuseettiset toimikunnat) |
| Instituto de Investigacion Hospital 12 de Octubre (i+12) |
| Instytut Pomnik Centrum Zdrowia Dziecka |
| Kantonale Ethikkommission Bern (KEK) |
| Kantonale Ethikkommission Zuerich |
| Klinika Medyczna Ibis Spzoo i Wspolnicy spolka komandytowa |
| Komisja Bioetyczna przy Okregowej Izbie Lekarskiej |
| Lithuanian Bioethics Committee |
| Local Ethic Committee University Multiprofile Hospital for Active Treatment Sveti Georgi EAD |
| Local Ethics Committee USHATED Academy Ivan Penchev |
| Lux Med Spzoo |
| Manitoba Clinic |
| Medical Plus sro |
| Melbourne Health Human Research Ethics Committee |
| Misericordia General Hospital |
| Multi Region Ethics Committee |
| Narodny onkologicky ustav |
| Narodny ustav reumatickych chorob Piestany |
| National Bioethics Committee for Medicines and Medical Devices |
| National Ethics Committee |
| Nemocnica Kosice-Saca as, I sukromna nemocnica |
| Nemocnice Ceske Budejovice as |
| Northern A Health and Disability Ethics Committee |
| Northern Sydney Central Coast Area Health Service Human Research Ethics Committee |
| NZOZ Krajmed |
| NZOZ Nasz Lekarz Praktyka Grupowa Lekarzy Rodzinnych z Przychodnia Specjalistyczna |
| NZOZ Osteo-Medic s.c A. Racewicz, J. Supronik |
| Osteologicke Centrum |
| Osteon Osrodek Leczenia Osteoporozy i Chorob Stawow |
| Quorum Review Inc. |
| Radiodiagnostika |
| Radiologie a zobrazovaci metody Mestska nemocnice Ostrava |
| Radiologie Varad |
| Regionala Etikprovningsnamnden i Uppsala |
| Regionale Komiteer for medisinsk forskningsetikk, Helseregion Midt-Norge |
| Research Review Board, Inc. |
| Revmatologicky ustav |
| Samodzielny Szpital Miejski im Polskiego Czerwonego Krzyza w Bialymstoku |
| Sanus Spzoo NZOZ Przychodnia Lekarska Obluze Lesne |
| Specjalistyczny Osrodek Medycyny Wieku Dojrzalego Spzoo |
| SPSK im prof Dr Witolda Orlowskiego Centrum Medycznego Ksztalcenia Podyplomowego |
| SPZOZ Wojskowa Specjalistyczna Przychodnia Lekarska |
| Synexus Polska Spzoo |
| Szpital Miejski nr 1 |
| Tallinn Medical Research Ethics Committee |
| Trent MREC |
| Universitair Ziekenhuis Gent - Ethisch Comite |
| Univerzitna nemocnica Bratislava - Nemocnica Ruzinov |
| Univerzitna nemocnica Bratislava - Nemocnica Stare Mesto |
| Uniwersytecki Szpital Kliniczny w Bialymstoku |
| Wojewodzki Specjalistyczny Szpital Dzieciecy im Swietego Ludwika w Krakowie |
